# The complete plastid genome of *Rhamnus taquetii*, an endemic shrub on the Jeju Island of Korea

**DOI:** 10.1080/23802359.2020.1719933

**Published:** 2020-01-29

**Authors:** Dong-Pil Jin, Jong-Won Park, Jong-Soo Park, Byoung-Hee Choi

**Affiliations:** Department of Biological Sciences, Inha University, Incheon, Republic of Korea

**Keywords:** Endemic species, Rhamnaceae, *Rhamnus taquetii*, plastid genome

## Abstract

*Rhamnus taquetii* (family Rhamnaceae) is a shrub , endemic to Korea. Here, the *R*. *taquetii* plastid genome was found to be 161,205 bp long and consists of quadripartite structures; a large single-copy region of 89,373 bp, a small single-copy region of 18,936 bp, and a pair of inverted repeat regions of 26,448 bp each. The GC content of the sequence was found to be 37.1%. The plastid genome was found to harbor 129 genes, including 84 protein-coding genes, 37 transfer RNA genes, and 8 ribosomal RNA genes. On the phylogenetic tree of Rosales (based on 63 protein-coding genes), Rhamnaceaewas found to be monophyletic.

The genus *Rhamnus* L. s.l. (Rhamnaceae) is composed of about 150 species, that range from shrubs to trees (Chen and Schirarend 2007), and occurs mainly in the tropical to temperate zones of the Northern Hemisphere (Chen and Schirarend 2007). *Rhamnus* has a complex taxonomic history, and several species within the genus have recently been considered as distinct genera (Hauenschild et al. 2016). Among seven *Rhamnus* species in Korea (Chang et al. 2011), *Rhamnus taquetii* (H. Lev.) H. Lev. is an endemic deciduous shrub, growing only on Jeju Island. This species is distinguished from other Korean *Rhamnus* species by the presence of spines at the terminal of branches, alternating leaves, and small stature (Chang et al. 2011). However, these features occasionally overlap with those of *Rhamnus parvifolia* Bunge and *Rhamnus rugulosa* Hemsl. (Chang et al. 2011), taxonomic position of *R*. *taquetii* has been controversial. Here, we report the complete plastid genome (plastome) of *R*. *taquetii*, which could help in the conclusive taxonomic classification of *R. taquetii.*

Leaves of *R. taquetii* were collected from Jeju Island, Korea (Voucher specimen: 33°21′N, 126°29′E, D. P. Jin & J. W. Park 1905248, KH: Korea National Arboretum). Genomic DNA was extracted using a Qiagen DNeasy Kit (QIAGEN, Seoul, Korea) and then sequenced on the Illumina MiSeq platform (Macrogen, Seoul, Korea). A total of 19,539,837 paired reads were produced and mapped onto the plastome of *Berchemia berchemiifolia* (Makino) Koidz. (GenBank: MG739656). Regions of this draft genome with <1000× coverage and four junctions between the large single-copy (LSC) region, small single-copy (SSC) region, and two inverted repeats (IRs), were verified using Sanger sequencing. Referencing the plastome of *Berchemia berchemiifolia*, genes of the draft genome were annotated using Geneious 7.1.8 (Biomatters Ltd., Auckland, NZ), but some genes were manually confirmed. Transfer RNAs (tRNAs) were confirmed using tRNAscan-SE (Lowe and Chan 2016). The plastome of *R*. *taquetii* (GenBank: MN901522) is 161,205 bp long and consists of an LSC (89,373 bp) region, an SSC (18,936 bp) region, and a pair of IRs (26,448 bp), with an overall GC content of 37.1% and a protein-coding region covering 76.0% of the plastome. The total number of genes in the plastome was 129 (83 protein-coding genes, 38 tRNA genes, and 8 ribosomal RNA genes). *Rhamnus taquetii* plastid gene content and arrangement closely coincided with those of *B. berchemiifolia* (Cheon et al. 2018), but the *psbL* gene of *R*. *taquetii* was determined as an intact gene.

The maximum-likelihood tree of Rosales was constructed based on 63 protein-coding genes with RAxML 8.2.11 (Stamatakis 2014) (Figure 1). GTR + G + I was selected as the nucleotide substitution model according to the Akaike Information criterion throughout jModelTest 2.1.6 (Darriba et al. 2012). *Lespedeza maritima* Nakai (Fabaceae) was used as the outgroup. On the phylogenetic tree (Figure 1), Rhamnaceae was supported as monophyletic, and *R*. *taquetii* was closely clustered with *Berchemia berchemiifolia* and *Berchemiella wilsonii* (C.K. Schneid.) Nakai (GenBank: MH938366). Rhamnaceae was a sister to Elaeagnaceae and Barbeyaceae, supporting the result of Zhang et al. (2011).

**Figure 1.**
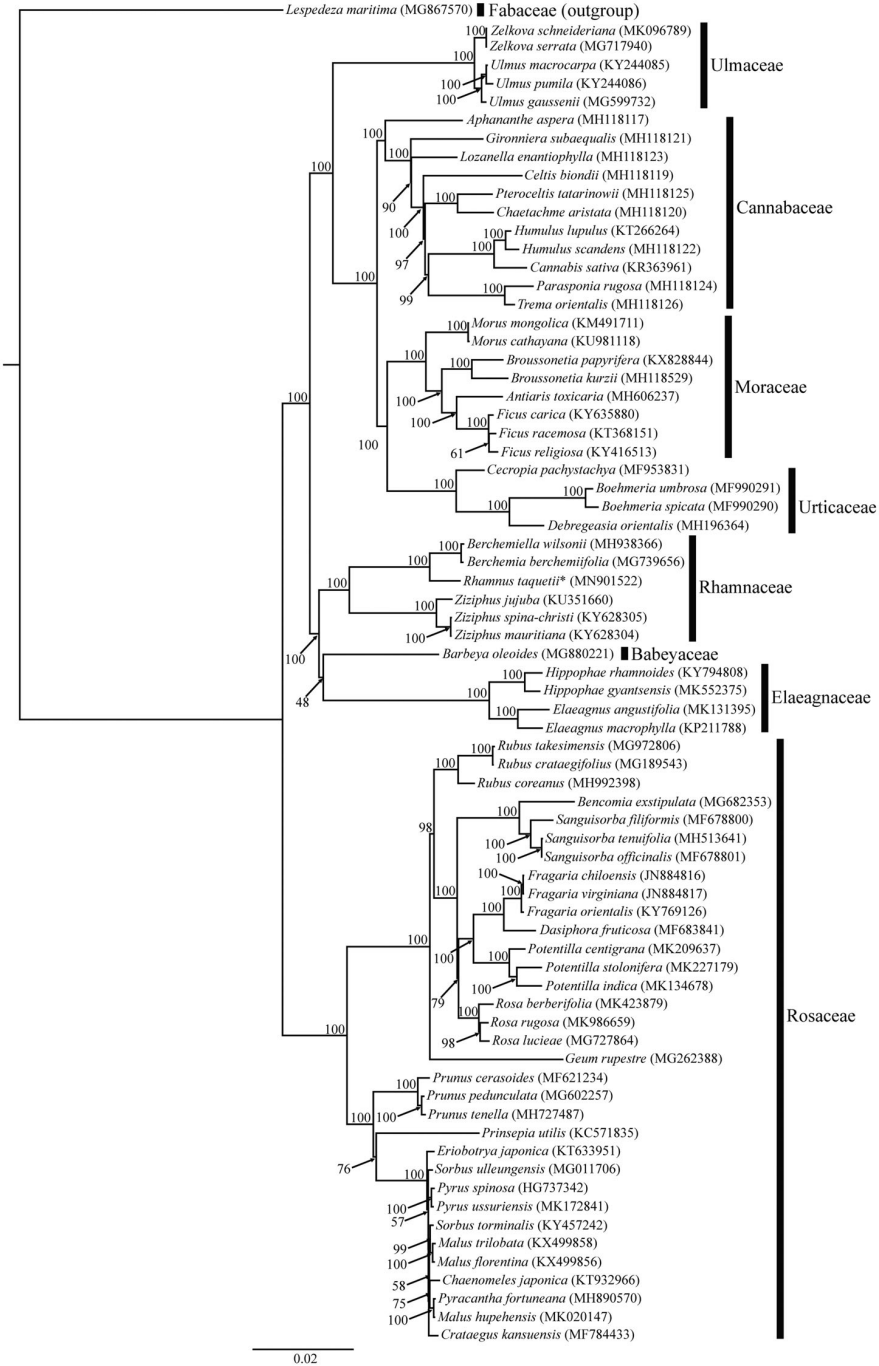
A maximum-likelihood tree of Rosales based on 63 coding-genes on plastid genomes. The NCBI accession number for each species is given after its scientific name. The number at each node indicates a bootstrap value. Taxon sequenced here is marked with an asterisk.
